# Presumptive Congenital Unilateral Renal Agenesis With Contralateral Dysplasia/Hypoplasia in a Golden Retriever

**DOI:** 10.1002/vms3.70973

**Published:** 2026-04-25

**Authors:** Hyewon Moon

**Affiliations:** ^1^ School of Veterinary Science The University of Queensland Gatton Queensland Australia

**Keywords:** canine, congenital abnormalities, kidney, renal agenesis, renal dysplasia, renal hypoplasia

## Abstract

**Background:**

Congenital renal agenesis with contralateral dysplasia/hypoplasia represents an exceptionally rare morphological presentation in dogs, with only one previous case documented worldwide.

**Case Description:**

A 4‐year‐old Golden Retriever (*Canis lupus familiaris*) presented with polyuria, polydipsia, inappetence and urinary incontinence. Laboratory findings revealed severe azotaemia, hyperphosphataemia and non‐regenerative anaemia.

**Results:**

Multimodal diagnostic imaging demonstrated complete presumptive left renal agenesis with a small dysplastic remnant and severe presumptive right renal dysplasia/hypoplasia with loss of normal renal architecture. The dog showed initial improvement with supportive care but experienced progressive deterioration, with euthanasia elected 18 days after presentation.

**Conclusions:**

This case documents an extremely rare morphological pattern of presumptive congenital renal abnormalities in a dog with an atypically late clinical presentation. The findings emphasise the diagnostic value of imaging in differentiating congenital renal disease from acute kidney disease and expand the current understanding of its potential clinical course in adult dogs.

## Graphical abstract

A 4‐year‐old Golden Retriever presented with polyuria, polydipsia and severe azotaemia, with multimodal imaging revealing complete presumptive left renal agenesis with a small dysplastic remnant and severe presumptive right renal dysplasia/hypoplasia with loss of normal corticomedullary architecture. To the authors’ knowledge, this represents the first documented case of this rare morphological combination in a Golden Retriever, a breed with known hereditary predisposition to renal dysplasia, and only the second reported case worldwide. The atypically late presentation at 4 years of age suggests greater functional compensation than previously recognised for this morphological variant.

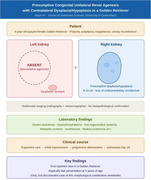

## 1 Introduction

Congenital renal agenesis is a rare developmental abnormality characterised by the complete absence of kidney formation during embryogenesis. The condition is associated with variations in cyclooxygenase‐2 (COX‐2) genetic expression, as demonstrated in canine and mouse models (Norwood et al. 2000; Whiteley 2014). Bilateral agenesis is fatal (Brownie et al. 1988), while unilateral cases depend on the compensatory function of the remaining kidney (Morita et al. 2005). Most reported cases of renal agenesis in dogs present with juvenile onset of clinical signs under 3 years of age (de Morais et al. 1996; Hoppe and Karlstam 2000; Kerlin and Van Winkle 1995). While unilateral renal agenesis as an isolated finding has been documented in several dog breeds (Agut et al. 2002; Robbins 1965; Taney et al. 2003), the combination of unilateral renal agenesis with contralateral renal dysplasia/hypoplasia is exceptionally rare in dogs; to our knowledge, only a single canine case has been reported, in a 3‐year‐old Cavalier King Charles Spaniel (Morita et al. 2005). This case describes presumptive unilateral renal agenesis with contralateral dysplasia/hypoplasia in a 4‐year‐old Golden Retriever, representing both the first documented case of this condition in the breed (Kerlin and Van Winkle 1995; Miyamoto et al. 1997) and an unusually late age of presentation, thereby providing valuable insight into the clinical manifestation, extended survival potential and disease progression of this rare morphological variant.

### 1.1 Patient Information

A 4‐year‐old spayed female Golden Retriever weighing 31.8 kg was presented to a veterinary hospital on 25 July 2025. The owner reported a progressive 2‐week history of marked polyuria and polydipsia, nocturnal urinary incontinence occurring during sleep, progressive inappetence and general lethargy.

The patient had an unremarkable medical history with no previous episodes of illness or hospitalisation. Regular preventive health care had been maintained throughout life, including annual wellness examinations, current core vaccinations, and year‐round heartworm prevention. Family history was unknown, and no genetic testing had been performed, limiting assessment of potential hereditary predisposition. The dog lived as a single pet in a suburban household with no known exposure to nephrotoxins or recent dietary changes. No previous medical interventions had been required, and all previous annual health assessments had been within normal limits.

### 1.2 Clinical Findings

Physical examination revealed a bright, alert and responsive Golden Retriever with appropriate body condition. Vital signs, including heart rate and respiratory rate, were within normal limits for age and breed. Hydration assessment showed pink and moist mucous membranes with a normal skin tent test, indicating no clinical evidence of dehydration. Cardiovascular examination demonstrated strong, synchronous peripheral pulses with cardiac auscultation revealing no murmurs, arrhythmias or abnormal heart sounds. Respiratory assessment showed clear lung sounds bilaterally with no adventitious sounds. Abdominal examination revealed a soft, non‐painful abdomen with no palpable masses, organomegaly or distension. Neurological assessment confirmed the patient was alert and responsive with normal mentation.

### 1.3 Timeline

The dog first presented on 25 July 2025 (Day 0) with polyuria, polydipsia and reduced appetite. Serum biochemistry confirmed severe azotaemia (creatinine 792 µmol/L). On Days 1–2, hospitalisation with intravenous fluid therapy resulted in short‐lived improvement. On Day 3, the dog was discharged with home medications and a renal diet. During Days 4–14, home management was pursued, but a gradual decline in appetite and activity occurred despite therapy. On Day 15, re‐evaluation documented persistently severe azotaemia (creatinine 780 µmol/L) with non‐regenerative anaemia; medical care continued with a palliative focus. On Day 18 (10 August 2025), humane euthanasia was elected due to progressive clinical deterioration and poor quality of life.

## 2 Diagnostic Assessment

### 2.1 Laboratory Testing

Haematological testing revealed non‐regenerative anaemia, with a packed cell volume ranging from 20% to 26% (reference interval: 33%–56%) and haemoglobin concentrations between 6.8 and 9.9 g/dL (reference interval: 11.0–19.0 g/dL). White blood cell and platelet counts were within normal limits.

Serum biochemistry demonstrated severe azotaemia, with creatinine concentrations of 662–792 µmol/L (reference interval: 50–127 µmol/L) and blood urea nitrogen ranging from 36 to 46 mmol/L (reference interval: 3.0–9.8 mmol/L). Marked hyperphosphataemia (2.54–4.84 mmol/L; reference interval: 0.80–1.70 mmol/L) and mild hypercalcemia (2.86–3.55 mmol/L; reference interval: 1.87–2.83 mmol/L) were present. Albumin was slightly reduced (27–29 g/L; reference interval: 28–44 g/L), while total protein remained within the reference interval.

Blood gas analysis confirmed severe metabolic acidosis, with pH values of 7.186–7.324 (reference interval: 7.35–7.45), bicarbonate concentrations of 12.4–16.5 mmol/L (reference interval: 20.0–24.0 mmol/L) and base excess ranging from −15.8 to −9.6 mmol/L (reference interval: −5.0 to 0 mmol/L).

Urinalysis was performed on a free‐catch urine sample and revealed isosthenuria (USG 1.012) despite significant azotaemia, consistent with renal insufficiency. Marked proteinuria (3+) was noted on dipstick analysis; urine protein‐to‐creatinine ratio was not performed. Microscopic evaluation identified numerous bacterial rods and neutrophils; however, as the sample was obtained by free catch and subsequent urine culture yielded no bacterial growth, a concurrent urinary tract infection could not be confirmed. Antibiotic therapy had been initiated prior to culture submission, which may have contributed to the negative culture result. The sediment was otherwise inactive, with no casts or significant haematuria.

### 2.2 Diagnostic Imaging

**FIGURE 1:**
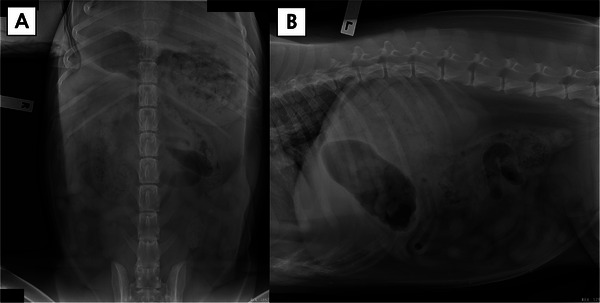
Abdominal radiographs. (A) Left lateral view demonstrating a single kidney in cranial abdominal position (right kidney) with an irregular shape. The left kidney is not radiographically visible. Good serosal detail is preserved. (B) Ventrodorsal view showing the misshapen right kidney visible on the right side of the abdomen. The left kidney is not radiographically evident. No mineral opacities suggestive of uroliths or nephroliths are detected.

Abdominal radiographs demonstrated the absence of a radiographically visible left kidney (Figure 1). On the left lateral view, a single kidney in cranial abdominal position was visible with an irregular shape, consistent with the right kidney (Figure 1A). On the ventrodorsal view, the misshapen right kidney was visible on the right side of the abdomen (Figure 1B). No mineral opacities suggestive of uroliths or nephroliths were detected, and overall serosal detail was preserved.

**FIGURE 2:**
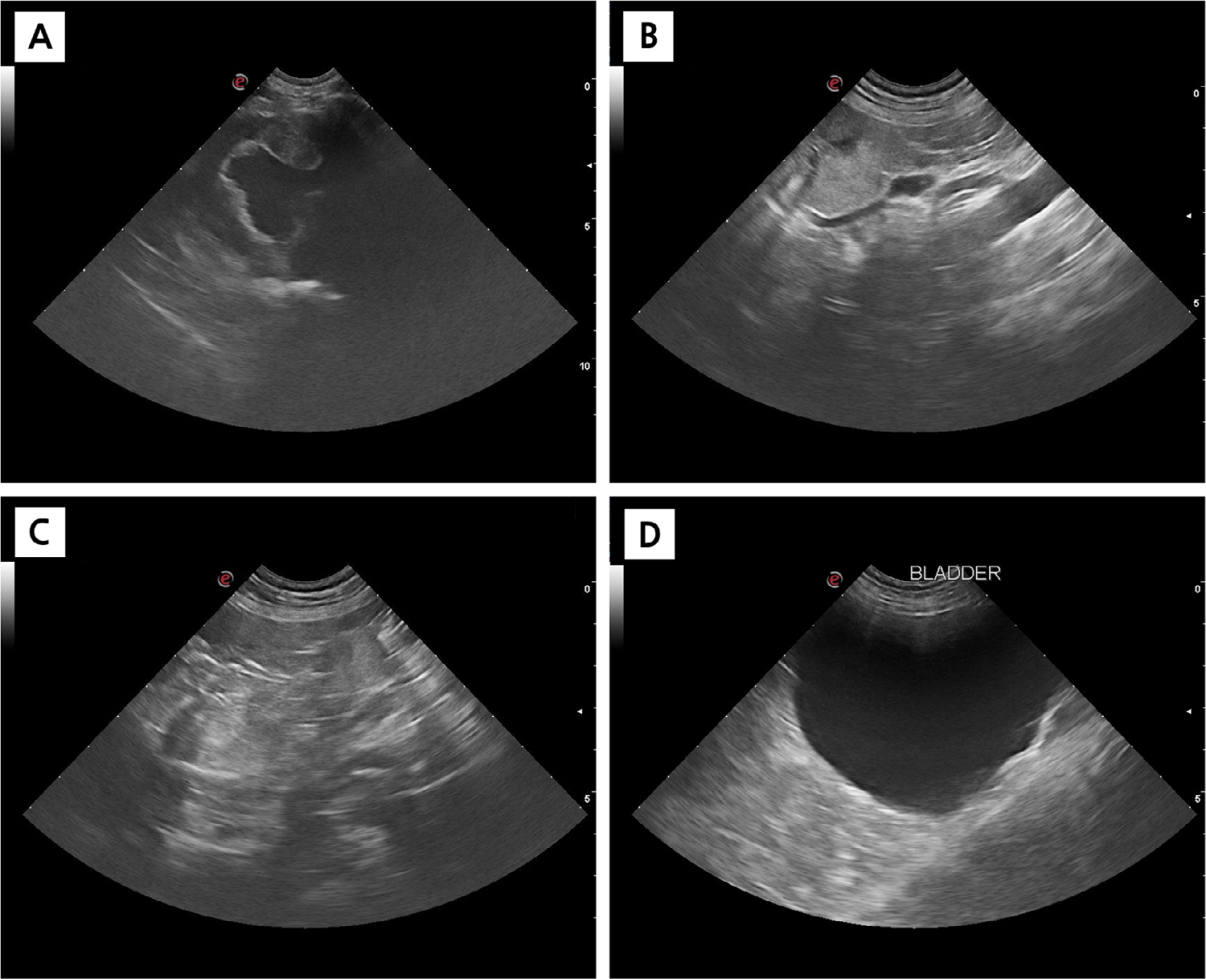
Abdominal ultrasonography of the urinary tract. (A) Left renal fossa showing a small dysplastic remnant (2.59 × 1.11 cm), with mixed echogenicity located adjacent to major blood vessels (aorta/caudal vena cava) caudal to the right kidney. (B) Longitudinal view of the dysplastic right kidney demonstrating reduced size (6.13 cm length), irregular margins, loss of normal corticomedullary architecture, mixed hyperechogenicity, and renal pelvic dilation (0.67 cm diameter). (C) Transverse view of the dysplastic right kidney showing heterogeneous parenchyma with occasional small cystic‐like structures in the outer cortical area. (D) Markedly distended urinary bladder with anechoic contents and normal wall thickness. All ultrasonographic images were obtained using a GE curved array transducer (mC 3–11, 3–11 MHz).

Abdominal ultrasonography revealed the absence of a normal left kidney (Figure 2). Instead, a small dysplastic remnant measuring 2.59 × 1.11 cm with mixed echogenicity was visualised caudal to the right kidney, situated adjacent to major blood vessels (Figure 2A). The right kidney was grossly abnormal, with reduced size (6.13 cm in length), irregular margins, loss of corticomedullary architecture, mixed hyperechogenicity, and renal pelvic dilation (0.67 cm diameter) demonstrated on longitudinal view (Figure 2B). Transverse view of the right kidney showed heterogeneous parenchyma with occasional small cystic‐like structures in the outer cortical area (Figure 2C). The urinary bladder was markedly distended with anechoic contents but exhibited normal wall thickness, trigone and proximal urethra (Figure 2D). No evidence of urolithiasis or free abdominal fluid was observed.

### 2.3 Therapeutic Intervention

Antibiotic therapy was initiated with amoxicillin (400 mg orally, three times daily for 14 days) for the management of a presumptive urinary tract infection. Anti‐emetic treatment included maropitant (1 mg/kg intravenously once daily) and ondansetron (0.2 mg/kg intravenously twice daily as required) to control nausea and vomiting. Supportive therapy consisted of intravenous administration of Hartmann's solution at 120–200 mL/h during hospitalisation to address dehydration, azotaemia and electrolyte derangements.

A prescription renal diet (Royal Canin Renal) was recommended to reduce phosphorus intake and support residual renal function.

Daily clinical monitoring was performed during hospitalisation, including repeated assessment of hydration status, mucous membrane colour, body weight, appetite and urination. Serial blood chemistry panels were obtained to monitor trends in creatinine, electrolyte concentrations and acid–base status.

### 2.4 Follow‐Up and Outcomes

Within 48 h of initiating intravenous fluid therapy, the patient exhibited a modest clinical improvement, with increased appetite and improved demeanour. Despite this, azotaemia showed minimal biochemical improvement, with creatinine decreasing only marginally from 792 to 780 µmol/L. The patient's condition stabilised sufficiently to allow discharge from the hospital.

Over the following 2 weeks, clinical status gradually declined despite continued medical management. The patient developed progressive inappetence, intermittent vomiting, and reduced quality of life. Humane euthanasia was elected 18 days after the initial presentation, and private cremation was arranged at the owners’ request.

Owner compliance with prescribed medications and dietary recommendations was excellent. The patient tolerated all treatments well, including repeated intravenous catheterisation and fluid therapy. Mild sedation was required for certain diagnostic procedures due to patient anxiety, but no significant adverse or unexpected events were reported during the treatment period.

## 3 Discussion

The primary strength of this case report lies in its documentation of the clinical course, multimodal imaging findings, and atypically late presentation of an exceptionally rare congenital renal morphological variant. Unlike typical cases of congenital renal disease that present as bilateral dysplasia/hypoplasia, this case demonstrates the rare combination of complete unilateral renal agenesis with contralateral dysplasia/hypoplasia. To our knowledge, this represents only the second documented instance of this specific morphological pattern in veterinary literature (Morita et al. 2005). The detailed documentation of clinical course, imaging findings, and disease progression over an 18‐day period contributes to the limited existing literature on this rare morphological variant.

The major limitation of this report is the absence of histopathologic confirmation. Renal biopsy or necropsy would have allowed definitive tissue characterisation and identification of specific dysplastic features, potentially clarifying the pathogenesis and prognosis. Previous studies have suggested that cases lacking immature glomerular lesions may exhibit slower disease progression, which could explain the extended survival and delayed clinical manifestation observed in this patient (Picut and Lewis 1987). Per current IRIS guidelines, both SDMA and urine protein‐to‐creatinine ratio are recommended for CKD staging and proteinuria substaging, respectively (IRIS 2023); the absence of these measurements represents a limitation in formal disease staging. Genetic testing for polycystic kidney disease was not performed, despite the presence of cystic‐like cortical structures on ultrasonography.

Ultrasonography findings were consistent with presumptive agenesis of the left kidney and presumptive dysplasia/hypoplasia of the right, supporting a congenital diagnosis. The renal pelvic dilation (6.7 mm diameter) exceeded normal values for dogs (D'Anjou et al. 2011), consistent with the severity of renal insufficiency and structural abnormalities. While most cases present within the first 2–3 years of life, both this case and the Morita case demonstrated later presentations (Morita et al. 2005), suggesting that the specific anatomic combination may permit partial functional compensation that delays clinical manifestation. The 4‐year survival observed here exceeds that of the previously documented case, which survived for 3 years without histologic evidence of immature glomeruli. Without histopathologic confirmation in the present case, definitive correlation between tissue characteristics and survival duration cannot be established. However, the eventual progression to end‐stage disease reflected the fundamental limitation of inadequate functional nephron mass.

Clinically, the presentation closely mimics acute kidney injury, underscoring the diagnostic challenge of distinguishing congenital chronic kidney disease from acute processes in young adult dogs. The marked azotaemia in an otherwise alert and well‐compensated patient initially raised suspicion for acute renal disease. However, several findings collectively supported a chronic congenital origin: non‐regenerative anaemia, severe hyperphosphataemia, ultrasonographic demonstration of bilaterally abnormal renal architecture, persistent isosthenuria and inactive urine sediment. Diagnostic imaging was essential for differentiating this case from acquired or acute disease processes.

This case demonstrates that congenital renal abnormalities can present greater morphological diversity and later onset than previously recognised, with the combination of unilateral agenesis and contralateral dysplasia/hypoplasia representing an extremely rare developmental pattern that may allow extended survival compared to bilateral dysplasia/hypoplasia. Veterinarians should maintain diagnostic suspicion for congenital abnormalities even in dogs presenting beyond typical juvenile ages, as initial compensatory mechanisms may mask underlying structural deficits. While diagnostic imaging enables presumptive diagnosis and appropriate case management, the irreversible nature of developmental renal defects ultimately limits therapeutic success regardless of supportive care quality, emphasising the importance of early recognition for realistic prognostic counselling and appropriate quality‐of‐life decisions.

## Author Contributions

**Hyewon Moon**: conceptualisation, investigation, writing – original draft, writing – review and editing, project administration, data curation, supervision, methodology.

## Funding

The author has nothing to report.

## Ethics Statement

This case report involved a client‐owned dog receiving standard veterinary care. All identifying information has been removed to protect patient confidentiality. No experimental procedures were performed, and ethical approval was not required for this retrospective clinical case report.

## Conflicts of Interest

The author declares no conflicts of interest.

## Data Availability

The clinical data that support the findings of this case report are available from the corresponding author upon reasonable request.
